# Wiskott-Aldrich syndrome protein restricts cGAS/STING activation by dsDNA immune complexes

**DOI:** 10.1172/jci.insight.132857

**Published:** 2020-09-03

**Authors:** Giulia Maria Piperno, Asma Naseem, Giulia Silvestrelli, Roberto Amadio, Nicoletta Caronni, Karla Evelia Cervantes-Luevano, Nalan Liv, Judith Klumperman, Andrea Colliva, Hashim Ali, Francesca Graziano, Philippe Benaroch, Hans Haecker, Richard N. Hanna, Federica Benvenuti

**Affiliations:** 1 International Centre for Genetic Engineering and Biotechnology, Trieste, Italy.; 2 Section Cell Biology, Center for Molecular Medicine, University Medical Center (UMC) Utrecht, Utrecht University, Utrecht, Netherlands.; 3 Institute Curie Laboratoire Immunité et Cancer — INSERM U932 Transport Intracellulaire et Immunité, Paris, France.; 4 Department of Infectious Diseases, St. Jude Children’s Research Hospital, Memphis, Tennessee, USA.; 5 Respiratory, Inflammation and Autoimmunity, MedImmune LLC, Gaithersburg, Maryland, USA.

**Keywords:** Cell Biology, Immunology, Dendritic cells, Innate immunity

## Abstract

Dysregulated sensing of self–nucleic acid is a leading cause of autoimmunity in multifactorial and monogenic diseases. Mutations in Wiskott-Aldrich syndrome protein (WASp), a key regulator of cytoskeletal dynamics in immune cells, cause autoimmune manifestations and increased production of type I IFNs by innate cells. Here we show that immune complexes of self-DNA and autoantibodies (DNA-ICs) contribute to elevated IFN levels via activation of the cGAS/STING pathway of cytosolic sensing. Mechanistically, lack of endosomal F-actin nucleation by WASp caused a delay in endolysosomal maturation and prolonged the transit time of ingested DNA-ICs. Stalling in maturation-defective organelles facilitated leakage of DNA-ICs into the cytosol, promoting activation of the TBK1/STING pathway. Genetic deletion of STING and STING and cGAS chemical inhibitors abolished IFN production and rescued systemic activation of IFN-stimulated genes in vivo. These data unveil the contribution of cytosolic self–nucleic acid sensing in WAS and underscore the importance of WASp-mediated endosomal actin remodeling in preventing innate activation.

## Abstract 2

Excessive innate cell responses in WASp-null cells depend on activation of the cGAS/STING pathway.

## Graphical abstract

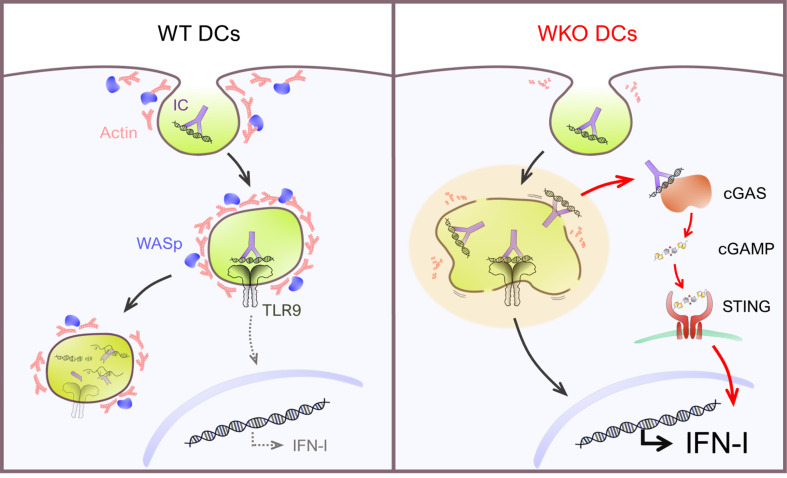

## Introduction

The link between innate sensing of endogenous nucleic acid (NA) and autoimmune manifestations has become clear in several monogenic diseases and in common autoimmune diseases such as systemic lupus erythematosus (SLE). Pathogenic production of type I IFN was initially linked to binding of endogenous NA or antimicrobial peptides to endosomal Toll-like receptors (1–3). More recent evidence suggests that the cytosolic cGAS/STING sensing system plays a critical role in recognition of self-DNA in systemic autoimmunity (4–6). cGAS resides in the cytosol and binds to dsDNA, catalyzing the synthesis of a cyclic dinucleotide (cGAMP), which in turn binds the adaptor STING and recruits TBK1 to activate IRF3 and type I IFN transcription (7, 8). In Aicardi-Goutières syndrome (AGS) and AGS animal models (4), dysregulated activation of the cGAS/STING pathway results from defects in the enzymes that degrade cytosolic DNA, such as the cytosolic exonuclease TREX1, leading to constitutive activation of IFN-stimulated genes (ISGs) and severe inflammation. Similarly, defects in lysosomal DNase II may lead to accumulation of undigested self-DNA in lysosomes, leakage to the cytosol, and severe autoinflammation in a cGAS/STING-dependent fashion (9–13). Moreover, partner proteins binding to self-DNA or alteration in endosomal maturation may promote leakage of ingested material into the cytosol, inducing activation of cytosolic innate pathways (14–17).

Wiskott-Aldrich syndrome (WAS) is a rare immunodeficiency caused by mutations in WAS protein (WASp), a key hematopoietic-specific actin nucleation-promoting factor (NPF) of the WASp/WAVE family. Autoimmune manifestations in WAS include the presence of elevated levels of antibodies to dsDNA and dysregulated production of inflammatory cytokines (18–24). Autoantibody production is driven by unrestrained activation of endosomal TLR9 and TLR7 (25, 26) in autoreactive B cells and by activatory signals from bystander cells such as platelets and neutrophils that contribute to exacerbate inflammation (23, 27). Recent evidence indicated that WASp myeloid cells contribute to autoimmunity by producing enhanced type I IFN and IL-1β (22–24). The major initial trigger and the sensing pathways involved in innate activation of myeloid cells remain unclear at present.

In this study we show that lack of WASp expression in DCs renders cells responsive to small doses of self-DNA in immune complex with autoantibodies (DNA-ICs), inducing activation of type I IFN genes. We further dissected the underlying cell biological mechanisms by showing that WASp controls endosomal actin and endolysosomal maturation, regulating disposal of ingested interferogenic material. Importantly, we propose that self-DNA accumulated within maturation-defective endosomes escapes into the cytosol, triggering the cGAS/STING pathway.

## Results

### WASp-deficient cells activate type I IFN genes upon stimulation with DNA-ICs.

**Figure 1:**
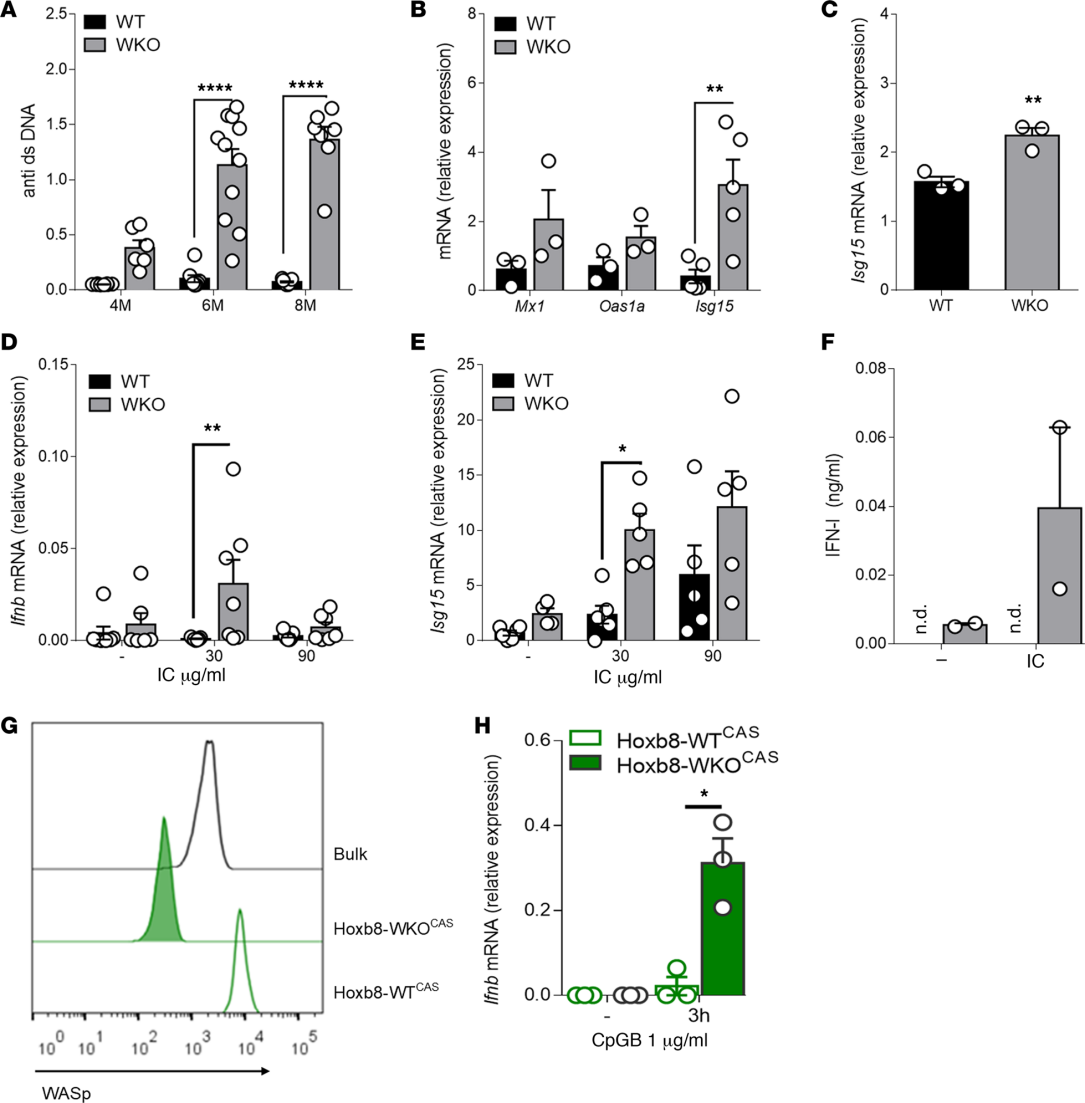
Enhanced type I IFN responses to endogenous DNA in Wasp-null DCs. (**A**) The content of anti-dsDNA in sera of WT and WKO animals was evaluated by ELISA at 4, 6, and 8 months of age. *n* = 8, 7, and 11 mice/group, respectively; mean ± SEM. ****P ≤ 0.0001, 2-way ANOVA. (**B**) Transcription of IFN-stimulated genes (*Mx1*, *Oas1a*, *Isg15*) in resting WT and WKO DCs. *n* = 3 for *Mx1* and *Oas1a* and *n* = 5 for *Isg15*; mean ± SEM. **P ≤ 0.01, 2-way ANOVA. (**C**) Transcription of *Isg15* in total resting splenocytes; *n* = 3 mice per group; mean ± SEM. **P ≤ 0.01, unpaired *t* test. (**D** and **E**) DCs were stimulated with the indicated doses of DNA-ICs (IC). Transcription levels of *Ifnb* (**D**; *n* = 7) and *Isg15* (**E**; *n* = 5) were evaluated by RT-PCR; mean ± SEM. *P ≤ 0.05, **P ≤ 0.01, 2-way ANOVA. (**F**) As in **D**, type IFN protein was evaluated by B16-Blue reporter assay; *n* = 3. n.d., not detected. (**G**) Histograms show WASp expression in genome-edited WT^CAS^ and WASp^CAS^ Hoxb8–derived DCs. (**H**) *Ifnb* gene transcription after CpG-B stimulation was evaluated by RT-PCR; mean ± SEM of 3 biological replicates. *P ≤ 0.05, unpaired *t* test. RT-PCR data are expressed as relative values normalized to GUSB.

To document the presence of reactivity to self-DNA in our cohort of WASp-null animals we collected sera at 3 different time points to analyze the presence of antibodies to dsDNA. The majority of animals developed autoantibodies after 4 months that further increased after 6 months and reached a plateau at 8 months (Figure 1A). In parallel, we observed active transcription of 3 ISGs (*Mx1*, *Oas1a*, and *Isg15*) in resting DCs and splenocytes of WASp-null animals, indicating ongoing responses to circulating interferogenic triggers (Figure 1, B and C). To directly link these observations, we prepared DNA immune complexes (ICs) of controlled stoichiometry mimicking that of endogenous complexes (28) to assess responses of WASp-null myeloid cells. DCs from WASp-KO (WKO) animals activated *Ifnb* gene transcription at the lowest concentration of DNA-ICs, whereas WT cells failed to respond to the highest dose of ICs tested (Figure 1D). Transcription of *Isg15* was also significantly enhanced in WKO cells at the lowest dose of immune-stimulatory DNA-ICs (Figure 1E), indicating activation of the downstream signaling cascade. Levels of bioactive protein confirmed enhanced production by WKO cells (Figure 1F). To further evaluate reactivity to synthetic DNA, we treated cells with CpG-B, which induces type I IFN through endosomal TLR9. *Ifnb* gene transcription and protein secretion were 3- to 4-fold higher in WKO DCs, at 2 different time points and doses (Supplemental Figure 1A; supplemental material available online with this article; https://doi.org/10.1172/jci.insight.132857DS1). Production of inflammatory cytokines (TNF-α, IL-12p40, and IL-6) was also enhanced in WKO DCs and in macrophages differentiated from WKO bone marrow precursors (BMDMs) (Supplemental Figure 1, B and C). To discriminate whether enhanced responsiveness to DNA-ICs is a cell-intrinsic property of WASp-null cells, we generated a WASp-deficient DC line (WKO^CAS^) by genome editing (29) (Figure 1G and Supplemental Figure 2A). Responses of WKO^CAS^ DCs were significantly greater than those of a control non-edited DC line (WT^CAS^), indicating that IFN-I levels are controlled by WASp in a cell-autonomous fashion (Figure 1H).

### Immune complexes stall in early compartments in WASp-KO cells.

**Figure 2:**
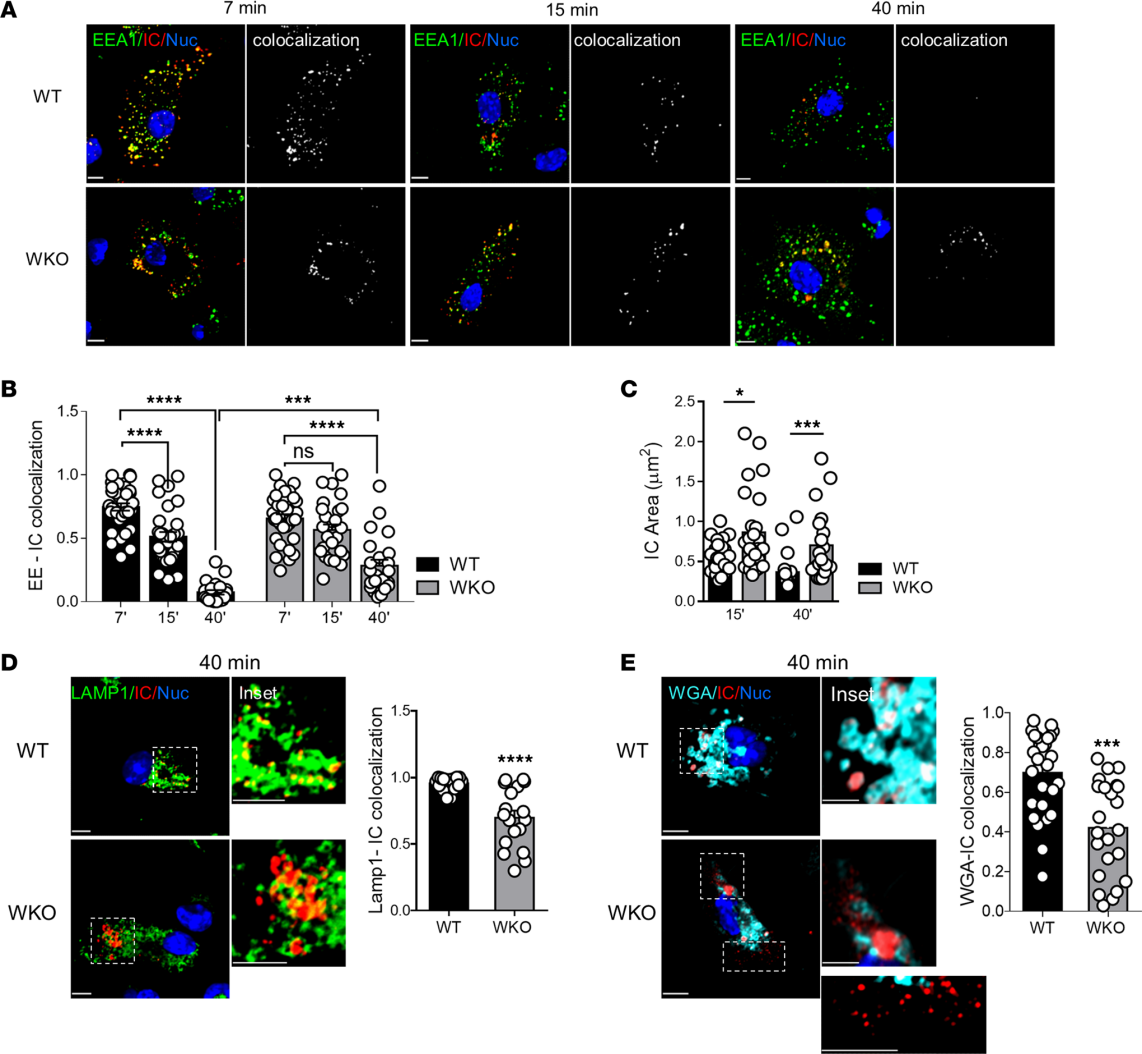
DNA-ICs accumulate in endosomes of WASp-null cells. (**A**) WT or WKO DCs were pulsed with DNA-ICs (90 μg/mL) for 7 minutes and chased for different durations. Cells were labeled with antibodies to the Fc portion of ICs and to early endosomes (EEA1). Images show representative confocal planes and the corresponding colocalization mask for early endosomes (EEs) and ICs. (**B**) Bars shows relative Mandel overlap coefficient (MOC) for ICs and EEs at the different time points. Mean ± SEM of 30 cells/condition from 3 independent experiments. ***P ≤ 0.001, **** *P* ≤ 0.0001, 2-way ANOVA. (**C**) The area of intracellular IC structures was measured 15 minutes or 40 minutes after pulse. Mean ± SEM. *P ≤ 0.05, ***P ≤ 0.001, unpaired *t* test, on 28 cells/condition from 2 independent experiments. (**D**) Images show representative central confocal planes of ICs and LAMP1 distribution after 40 minutes of chase. Insets are magnifications of the regions indicated by dotted lines. Graph shows MOC of ICs and Lamp1. Mean ± SEM of 28 cells/condition from 2 independent experiments. ****P ≤ 0.0001, unpaired *t* test. (**E**) DC were preloaded with labeled WGA (WGA-AF647) to mark lysosomes and incubated with DNA-ICs (90 μg/mL). Representative Airyscan micrographs and the corresponding insets show the relative distribution of ICs with respect to the WGA signal. The graph shows MOC of ICs and WGA (mean ± SEM of 28 WT and 23 WKO cells from 2 independent experiments). ***P ≤ 0.001, unpaired *t* test . Scale bars: 5 μm.

To explore the mechanism of enhanced reactivity of WASp-null cells, we tracked intracellular trafficking of DNA-ICs upon internalization. Seven minutes after pulse, most ICs were associated with early endosomes in both WT and WKO cells. At 15 and 40 minutes, we observed a gradual decrease in the overlap between ICs and EEA1 in WT cells, indicating exit from early compartments. The kinetics was delayed in WKO DCs, as ICs still overlapped with EEA1 at 15 and 40 minutes after pulse (Figure 2, A and B). The mean area of IC-positive structures was significantly larger in WKO cells after 15 minutes of pulse and increased even more after 40 minutes, indicating accumulation of cargo in early endocytic vesicles (Figure 2C). After 40 minutes, intracellular ICs almost completely overlapped with LAMP1 in WT cells. In contrast, WKO cells contained several large clusters of ICs that had not yet colocalized with LAMP1 (Figure 2D). To further examine delivery of ICs to lysosomes, cells were preloaded with WGA–Alexa Fluor 647 (WGA-AF647), which labels lysosomes (30). WGA-loaded DCs were exposed to ICs for 40 minutes, and their localization was analyzed by high-resolution microscopy. Most ICs clustered in the perinuclear area and overlapped with WGA in WT cells. In contrast, WKO cells contained several small and peripheral IC vesicles segregated from WGA organelles plus a few large IC structures only partially overlapping with WGA, which resulted in a significantly lower overlap coefficient (Figure 2E). Delayed intracellular degradation of ICs was confirmed by blotting of WT and WKO lysates at different time points after pulsing (Supplemental Figure 3A). Together these data show that transit of ingested cargo across endosomes of WASp-null cells is delayed, increasing the instantaneous concentration of immunogenic cargo in signaling organelles.

### WASp recruited on endosomes drives F-actin polymerization and controls endolysosomal architecture and function.

**Figure 3:**
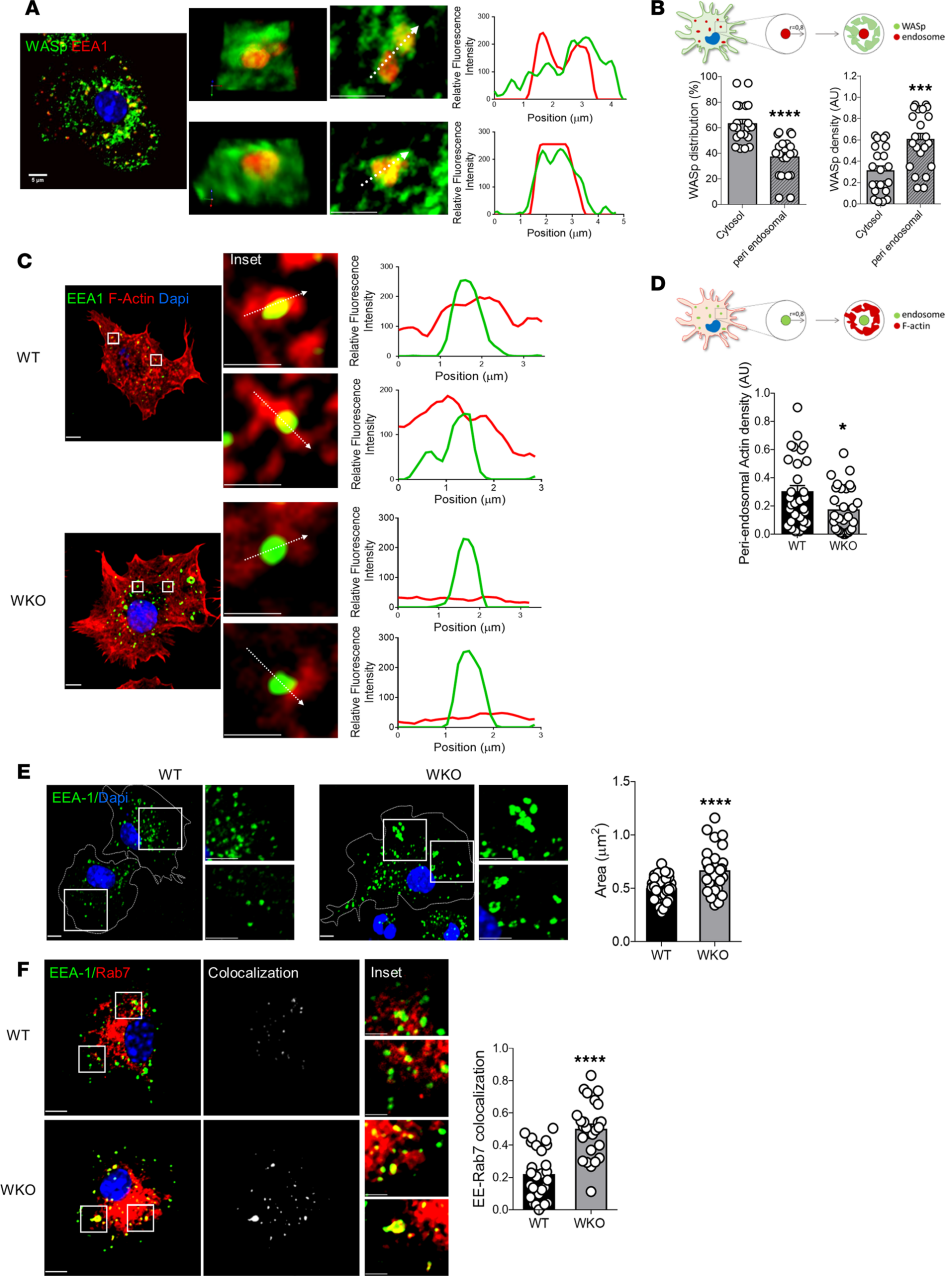
WASp controls endosomal F-actin nucleation and endosome architecture. (**A**) Representative high-resolution image of WASp distribution around endosomes (scale bar: 5 μm). Insets show magnification of the area indicated by the squares (scale bars: 2.5 μm). Right: Plot profiles of relative fluorescence intensity in the green and red channels along the dashed arrows indicated in the inset. (**B**) WASp distribution and density around endosomes were defined by computing a peri-endosomal radial line of 0.8 μm. Bars show the relative fraction of total WASp (left) and the density of WASp signal (right) in cytosol and around endosomes. Data were calculated automatically on 30 cells from 3 independent experiments. Mean ± SEM. ***P ≤ 0.001, ****P ≤ 0.0001, unpaired *t* test. (**C**) WT and WKO DCs were labeled with antibodies to EEA1 and F-actin. Single confocal planes for each genotype are shown; scale bar: 5 μm. Insets are zoom on individual endosomes corresponding to the indicated squares; scale bars: 1.5 μm. Right: Plot profiles of relative fluorescence intensity in the green and red channels along the indicated dashed arrows. (**D**) As above, the scheme shows the pipeline to quantify F-actin density. The density of peri-endosomal F-actin in WT and WKO DCs, calculated on all endosomes of 30 cells/condition from 3 independent experiments. Graphs show mean ± SEM. *P ≤ 0.05, unpaired *t* test. (**E**) Images are single confocal planes of WT and WKO DCs labeled with antibodies to EEA1. Insets are magnification of the area indicated by the squares (scale bars: 5 and 2 μm). Endosomal area was calculated on at least 30 cells/condition (mean ± SEM). ****P ≤ 0.0001, unpaired *t* test, from 2 independent experiment. (**F**) Single confocal planes showing EEA1 and Rab7 distribution and the corresponding colocalization mask (white). Insets show magnification of the area indicated by the squares. The overlap (MOC) between EEA1 and Rab7 is plotted. Mean ± SEM of at least 30 cells/condition from 2 independent experiments. ****P ≤ 0.0001, unpaired *t* test. Scale bars: 5 μm.

NPFs of the WASp family control endosomal dynamics by directly promoting endosomal F-actin nucleation in nonhematopoietic cells (31). To understand whether WASp may act as a regulator of endosomal F-actin in DCs, we first analyzed its intracellular distribution with respect to endosomes using high-resolution microscopy. A large fraction of WASp was localized around endosomes, forming a dense coat around endocytic vesicles (Figure 3, A and B). To verify whether WASp accumulation affected F-actin polymerization on endosomes, we measured its density in the peri-endosomal area. Interestingly, the density of endosomal F-actin was significantly diminished around WKO endosomes (Figure 3, C and D), indicating a direct role for WASp in nucleating endosomal actin. The decrease in endosomal F-actin correlated with an altered morphology, leading to enlarged endosomal area, irregular shape, and peripheral distribution in WKO cells (Figure 3E and Supplemental Figure 4A). Ultrastructural analysis of DCs that had taken up BSA-Au5 confirmed that area, perimeter, and diameter were significantly greater in WKO endolysosomes (Supplemental Figure 4B). Endosomal progression entails acquisition of specific membrane proteins that mark progressive maturation steps. Interestingly, analysis of specific markers of early (EEA1) and late endosomes (Rab7) showed defective segregation in WASp-null cells, suggesting loss of organelle identity (Figure 3F). By dynamic imaging of endocytic vesicles, we observed accumulation of large and poorly motile vesicles in WKO cells, confirming altered mobility and progression of endocytosed cargo (Supplemental Videos 1 and 2 and Supplemental Figure 4C).

Expression of the other members of the WASp/WAVE family was not modified in WKO DCs, ruling out that endosomal disorganization may depend on unbalanced expression of other NPFs (Supplemental Figure 5A). To confirm that endosome size is controlled by WASp-mediated actin dynamics, we treated cells with inhibitors of actin polymerization (latrunculin A [LAT-A]) or with the specific inhibitor of the Arp2/3 actin-nucleating factor (CK666), the downstream WASp effector. Both inhibitors induced a significant increase in the size of early endosomes (mean ± SD area of vehicle-treated cells, 0.53 ± 0.15 μm^2^; CK666-treated cells, 0.72 ± 0.08 SD μm^2^; LAT-A–treated cells, 1.05 ± 0.2 SD μm^2^) (Supplemental Figure 5B), suggesting that endosomal architecture is perturbed by decreased actin polymerization by WASp rather than other WASp-mediated pathways. We further investigated the impact of WASp-mediated Arp2/3 activation in cells treated with the CK666 inhibitor. The drug induced a delay in DNA-IC progression (Supplemental Figure 5, C and D) and rendered cells responsive to DNA-ICs (Supplemental Figure 5E), confirming that DNA-IC trafficking and innate responses are regulated by WASp via its actin-modulatory properties.

**Figure 4:**
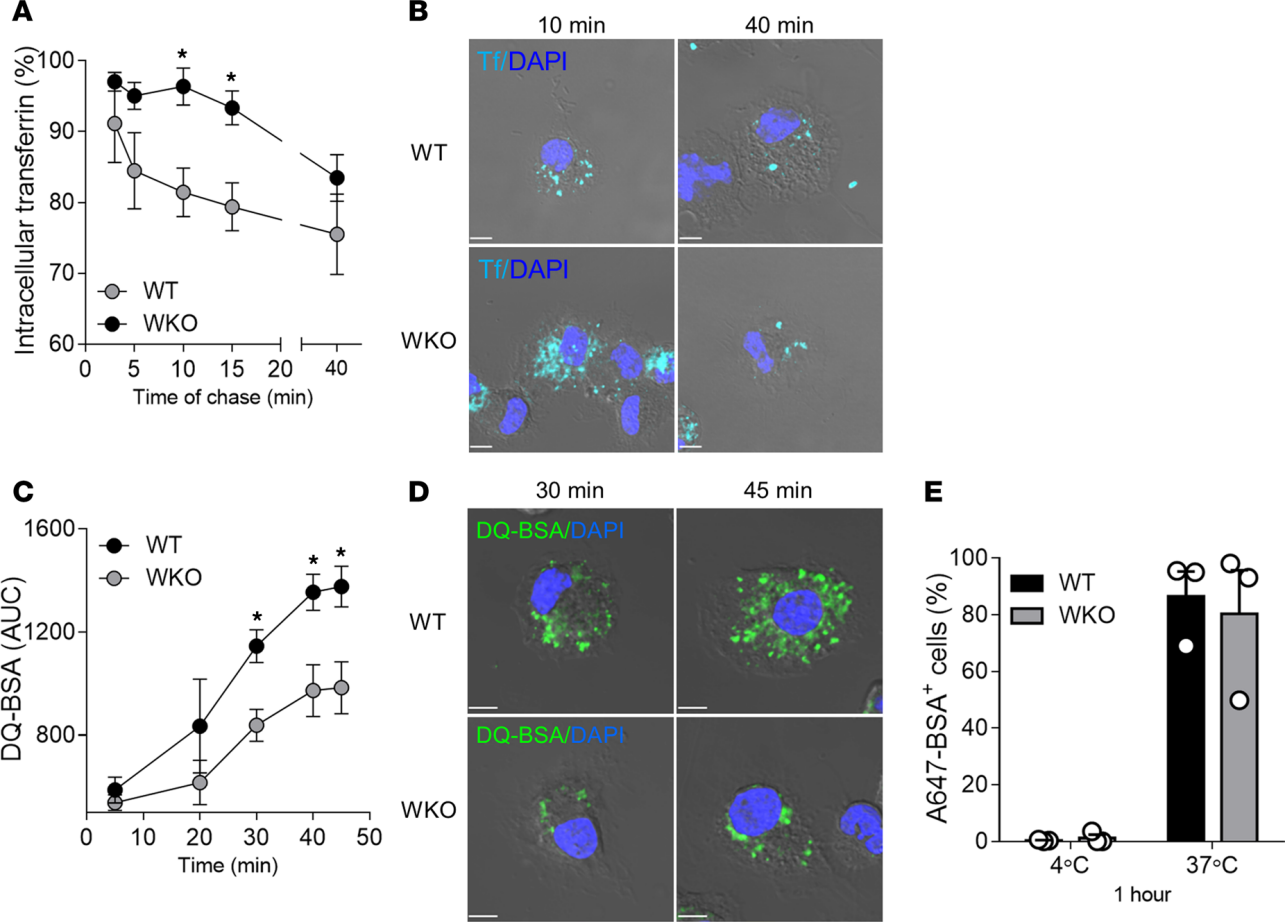
WASp controls endosomal recycling and lysosomal degradation. (**A** and **B**) WT and WKO DCs were loaded with AF647-Tf at 37°C for 1 hour, acid washed (*t* = 0), and incubated at 37°C for the indicated times. (**A**) Recycling is expressed as the percentage of intracellular Tf (**A**) remaining at the different time points with respect to *t* = 0. Mean ± SEM of 3 independent experiments. *P ≤ 0.05, unpaired *t* test. (**B**) Representative confocal plane of WT and WKO DCs at 2 different point of chase. (**C**–**E**) WT and WKO DCs were pulsed with FITC/DQ-BSA and AF647-BSA (as control for uptake) for 10 minutes at 37°C. Samples were washed and continuously acquired for 45 minutes on a flow cytometer. (**C**) Data represent AUC, normalized on AF647-BSA uptake (mean ± SEM of 3 independent experiments). *P ≤ 0.05, unpaired *t* test. (**D**) Images are a representative confocal plane of WT and WKO DCs at 2 points of chase after DQ-BSA pulsing. (**E**) Bars indicate the fraction of DCs positive for AF647-BSA after 1 hour at 4°C or 37°C, as a control for uptake between the genotypes. Scale bars in all images: 5 μm.

Morphological changes in WASp-null cells correlated as well with altered endosomal function. Measurement of recycling of labeled transferrin (Tf) indicated that recycling started after 5–10 minutes and reaches a plateau after 40 minutes in WT cells (mean ± SD, 24.5% ± 9.8% of recycled Tf). In contrast, no recycling was observed for the first 10–15 minutes in WKO DCs and only 16.5% ± 5.6% of Tf had recycled by the end of the assay, indicating a significant delay in the process (Figure 4A). Microscopy images captured at 2 time points confirmed more abundant intracellular Tf vesicles in WKO cells at early time points (10 minutes of chase) and a normalization at plateau (40 minutes of chase) (Figure 4B). Second, we assessed maturation into lysosomes by loading cells with dye-quenched BSA (DQ-BSA), a self-quenched probe that emits bright fluorescence only upon proteolytic cleavage. AF647-BSA, a degradation-insensitive probe, was used as a control for uptake. Fluorescence intensities (gated on cells positive for AF647-BSA) increased steadily from 20 to 45 minutes at 37°C, with a similar trend in both genotypes, but intensities were significantly higher in WT cells as compared with WKO cells at most time points tested (Figure 4C). Higher intracellular fluorescence in WT cells was confirmed by confocal microscopy (Figure 4D*)*. Of note, uptake of Alexa Fluor 647 BSA was similar in WT and WKO cells (Figure 4E), indicating that WASp deficiency does not affect internalization but only subsequent steps of delivery toward degradative compartments.

We conclude that a large fraction of intracellular WASp localizes to endosomes, where it contributes to regulation of the pool of endosomal F-actin and endosomal architecture, which is critical for organelle shape and identity and for trafficking of endocytosed cargo for recycling and degradation.

### Aberrant endosomes promote leakage of internalized cargo in the cytosol.

**Figure 5:**
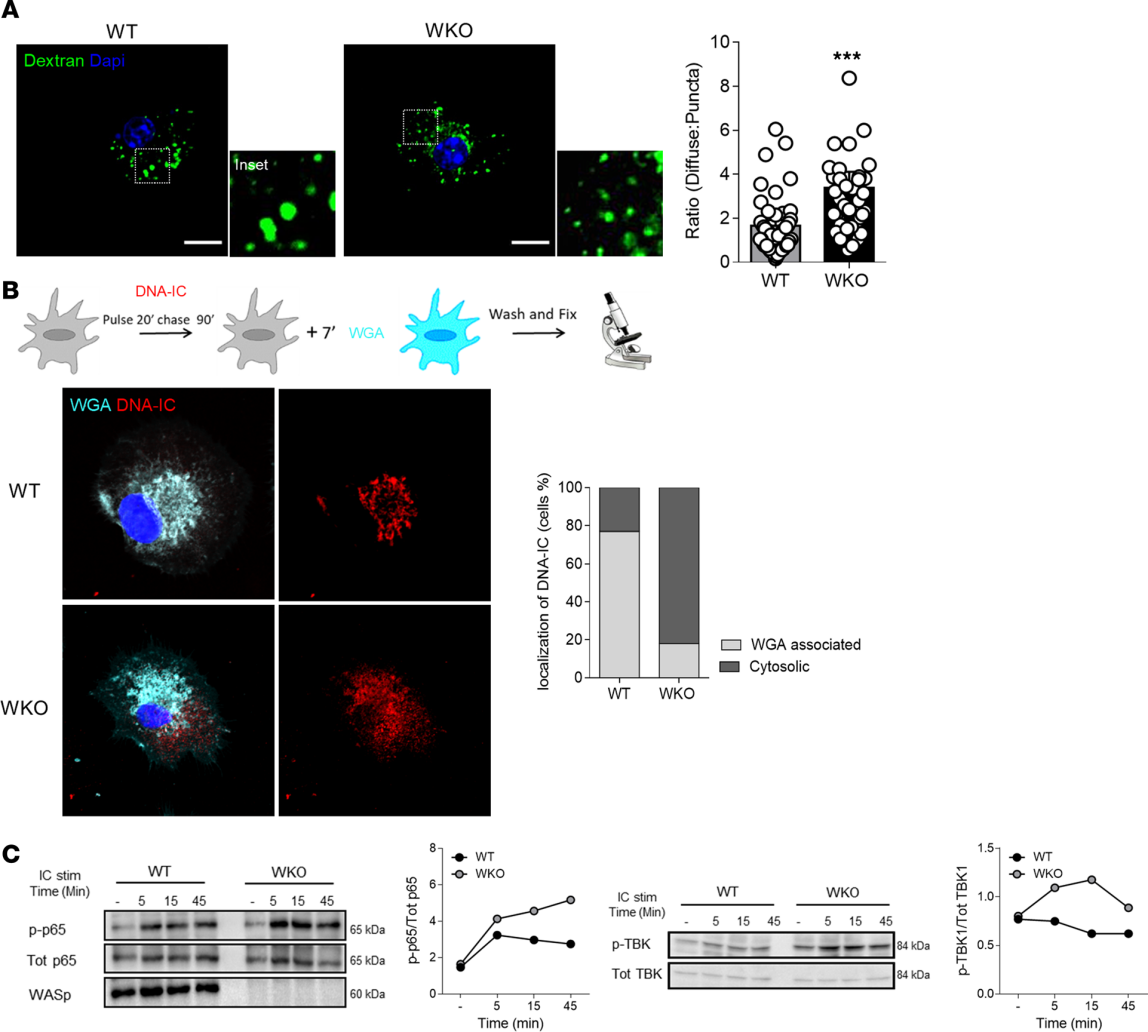
Defective endolysosomes leak to the cytosol, causing TBK1 activation. (**A**) Representative confocal images of DCs loaded with AF488-dextran (10 kDa) for 20 minutes (scale bar: 10 μm). Insets show magnification of the area indicated by the squares. The relative distribution of dextran signal within vesicular structures or in the cytosol was quantified in 45 individual cells from 3 independent experiments and expressed as diffuse/punctate ratio (mean ± SEM). ***P ≤ 0.001, unpaired *t* test. (**B**) DCs were pulsed with prelabeled DNA-ICs for 20 minutes, chased for 90 minutes, and counterstained with WGA-AF647 to identify the endocytic compartment. Images show representative Airyscan confocal sections of WGA and DNA-IC distribution. The fraction of cells showing DNA-IC signal within endosomes or outside endosomes (cytosolic) was calculated on 20 cells/condition from 2 independent experiments. (**C**) Lysates of WT and WKO DCs stimulated with DNA-ICs (IC stim; 90 μg/mL) were probed for p-p65, total p65 (Tot p65), p-TBK1, total TBK1, and WASp. p65 phosphorylation and TBK1 phosphorylation were normalized to their total form by Image Lab software (Bio-Rad) (1 representative of 3 experiments).

Stalling of ICs within WKO endosomes increases the concentration of ligand available for triggering of endosomal receptors, in line with our previously published data showing enhanced TLR9 activation (22). We speculated that in addition to altering endosomal signaling, defective endosomal maturation may also compromise membrane integrity and increase permeability to the cytosol (32). To test this hypothesis, we pulsed WT and WKO cells with a small-size labeled dextran (10 kDa). Dextran confined in vesicular structures was discriminated from dextran that had escaped into the cytosol based on decreased intensity, smaller spot size, and irregular shape using a semiautomated script (described in Methods). Indeed, the fraction of dextran diffused in the cytosol was significantly higher in WKO cells as compared with WT cells, indicating increased permeability of the endocytic compartment (Figure 5A). We next pulsed cells with prelabeled ICs and we analyzed their intracellular distribution with respect to endocytic membranes (marked with a short pulse of WGA staining). Ninety minutes after the pulse, the remaining IC signal overlapped entirely with endolysosomes in WT cells. In contrast, the IC signal in WKO cells was diffused in an area devoid of endolysosomal membranes, indicating cytosolic localization (Figure 5B).

### DNA-ICs induce type I IFN production via the STING pathway.

**Figure 6:**
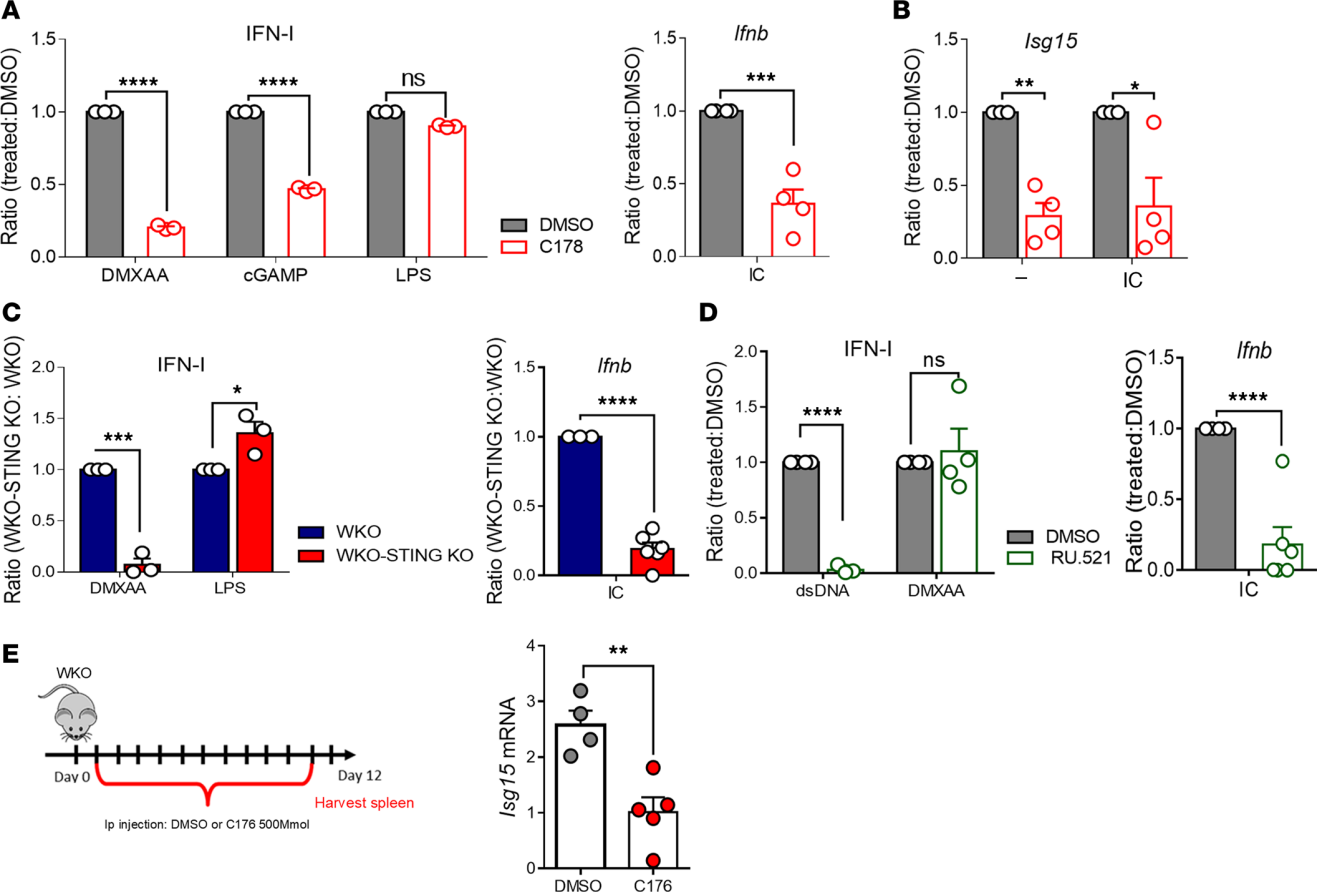
The cGAS/STING pathway controls responses to DNA-ICs in WASp-null cells. (**A**) WKO DCs were treated with C-178 (0.25 μM) or vehicle (DMSO) and stimulated with DMXAA (5 μg/mL), cyclic di-GMP (cGAMP, 3 μg/mL), LPS (1 μg/mL), and DNA-ICs (IC, 90 μg/mL). Type I IFN protein (for DMXAA, cGAMP, and LPS) or transcripts (for DNA-ICs) were evaluated by B16-Blue reporter assay or RT-PCR, respectively. Data are expressed as ratio between inhibitor-treated and DMSO-treated cells. Significance was determined by. Mean ± SEM of 3–4 biological replicates. ****P ≤ 0.0001, ***P ≤ 0.001, 2-way ANOVA for protein and by unpaired *t* test for transcript. (**B**) Transcription level of *Isg15* in resting or stimulated (DNA-IC, 90 μg/mL) WKO DCs that had been pretreated with C-178 (0.25 μM). Mean ± SEM of 4 biological replicates. *P ≤ 0.5, **P ≤ 0.001, 2-way ANOVA. (**C**) WKO and WKO STING-KO DCs were stimulated with DMXAA (5 μg/mL), LPS (1 μg/mL), and DNA-ICs (90 μg/mL). Type I IFN protein (DMXAA and LPS) or transcripts (DNA-ICs) are expressed as ratio between values in WKO STING-KO and values in WKO. Significance was determined by. Mean ± SEM of 3–6 biological replicates. *P ≤ 0.5, ***P ≤ 0.001, ****P ≤ 0.0001 2-way ANOVA for protein and unpaired *t* test for transcript. (**D**) WKO DCs were treated with RU.521 (50 μM) or vehicle (DMSO) and stimulated with dsDNA (2.5 μg), DMXAA (5 μg/mL), and DNA-ICs (90 μg/mL). Values were measured and expressed as in **A**. (**E**) WKO mice were treated for 11 consecutive days with 500 mM C-176 or DMSO. Total splenocytes were harvested after 12 days, and the levels of *Isg15* mRNA were evaluated by RT-PCR. Data are from *n* = 5 C-176 and *n* = 4 DMSO animals. Significance was evaluated by *t* test

To explore whether escape from endosomes can activate cytosolic sensors, we analyzed proximal signaling upon DNA-IC stimulation. Beside enhanced activation of NF-κB, WKO cells showed clear activation of TBK1, the kinase recruited by STING to induce type I IFN (Figure 5C) (33). WASp-null cells responded normally to direct STING stimulation (DMXAA, Supplemental Figure 6A), indicating an intrinsically normal pathway. To examine the actual contribution of STING to DNA-IC–induced IFN-I production, we first used the specific inhibitors C-176 and C-178 (34). Titration experiments identified the minimal working concentration of DMXAA (0.25 μM) used in subsequent experiments (Supplemental Figure 6B). C-178 specifically blocked phosphorylation of TBK1 upon DMXAA, confirming its specificity (Supplemental Figure 6C). As expected, C-178 inhibited responses induced by bone fide activators of the STING pathway, whereas it had no effect on LPS stimulation (Figure 6A). Importantly, C-178 completely inhibited type I IFN production and transcription of ISGs induced by DNA-ICs in WKO cells (Figure 6, A and B). To reinforce this finding and exclude possible nonspecific effects, we next deleted STING expression by CRISPR/Cas9 genome editing in WKO immortalized precursors (Supplemental Figure 6, D and E). Double-KO cells were differentiated into DCs (WKO-STING KO) from genome-edited precursors and stimulated with control agonists and DNA-ICs. Genome-edited cells showed the expected specific functional deactivation of the STING pathway and intact responses to unrelated pathways (Figure 6C). STING deletion abrogated *Ifnb* gene transcription upon DNA-IC stimulation (Figure 6C), confirming that DNA-IC–induced responses are driven by STING-dependent signaling. To further explore the pathway of DNA sensing in WKO cells, we interfered with cGAS activity using the specific RU.521 inhibitor. Stimulation of inhibitor-treated cells with dsDNA and STING agonist confirmed the efficacy and specificity of the treatment (Figure 6D). cGAS inhibition significantly reduced *Ifnb* and *Isg15* transcription (Figure 6D and Supplemental Figure 6F) induced by DNA-ICs, suggesting the involvement of cGAS in innate responses to self-DNA in WKO cells.

Finally, we explored the impact of inhibiting the STING pathway on constitutive activation of the IFN signature in vivo. Animals were repeatedly injected with C-176, a soluble variant of C-178 suitable for in vivo delivery (34). Organs were harvested after 12 days of treatment to assess the levels of *Isg15* in the spleen. Treatment with C-176 induced a mild but significant reduction in constitutive gene transcription in treated animals as compared with vehicle-treated WKO animals (Figure 6E).

We conclude that activation of the cGAS/STING pathway of innate sensing contributes to excessive production of type IFN-I in WASp-deficient cells and to chronic activation of the type I IFN pathway in vivo.

## Discussion

The mechanisms leading to erroneous recognition of self-DNA and loss of negative regulation of type I IFNs in autoimmune diseases are still not entirely understood and often disease specific. Here we reveal that a key contribution to pathogenic activation of type I IFN in WAS is the improper degradation and cytosolic leakage of ingested self-DNA. Altered endosomal dynamics in WASp-null cells affect the time of persistence of ingested cargo, lowering the threshold for activation of endosomal receptor. Second, decreased endosomal F-actin undermines membrane integrity and facilitates escape of internalized NA into the cytosol, leading to ectopic activation of the cGAS/STING pathway. These findings contribute to understanding unbalanced immune responses in WAS and suggest the potential benefit that inhibitors of the cGAS/STING pathway may have in controlling chronic innate activation.

In WAS, autoreactive B cells produce high levels of anti-dsDNA because of cell-intrinsic hyperreactivity of the B cell receptor and TLRs (18, 25, 35). These autoantibodies form complexes with dsDNA and represent a potential noxious trigger that boosts autoinflammation and sustains type I IFN levels. The data presented here show that a tiny amount of DNA-ICs, ignored by WASp-proficient cells, induce activation of type I IFN and ISGs in WASp-deficient cells in a cGAS/STING-dependent manner. Notably, administration of STING inhibitors in vivo rescued chronic transcription of Isg15 in the spleen of WASp-null animals, indicating that chronic systemic activation by circulating endogenous inducers depends on cytosolic sensing and can be modulated by inhibiting this pathway. Thus, as has been shown in monogenic disorders and in complex systemic diseases (4, 5, 36–38), cytoskeletal defects can impact the threshold of self-NA sensing.

To understand the molecular basis of increased sensitivity, we have extensively investigated endosomal architecture and function and we have followed the intracellular journey of DNA-ICs in WASp-null cells. WASp recruitment on endosomes coincident with actin enrichment suggest that hematopoietic WASp may work in a manner similar to the Arp2/3 activator WASH in nonhematopoietic cells, promoting endosomal progression and maturation (31, 39). In addition, WASp is part of a complex that bridges the actin and microtubule cytoskeleton via the adaptor CIP4 (40), affecting centripetal transport of vesicles (41), which is a further possible explanation for the delayed endosomal maturation found in WASp-null cells . An important accompanying feature of aberrant endosome dynamics that emerged in this study is increased endosomal membrane permeability. Indeed, endosomal leakage of accumulated ingested DNA or escape by active destabilization has been recently proposed to underlie excessive IFN responses in various conditions (10, 14, 42). Endosomal escape of engulfed DNA is facilitated by partner proteins such as HMGB1 and LL-37 that are secreted during cellular damage and are present in neutrophil extracellular traps (NETs) (15, 43). Although we have not evaluated the presence of HMGB1 in WAS, we speculate that HMGB1 levels may be elevated given the presence of NETs (23) and increased ROS activity (44), providing additional help to allow exit of DNA-ICs from stalling endosomes. Moreover, WASp-null cells show impaired autophagic flux following TLR4 and bacterial stimulation, a further factor known to predispose to membrane permeability (24, 45).

Our findings also contribute to explaining previous findings in WASp-deficient DCs. Delayed maturation of endosomes and escape to the cytosol are consistent with enhanced cross-presentation of ingested antigens in WASp-null cells (44), as this would favor slower degradation of class I epitopes and access to the endoplasmic reticulum for MHC class I peptide loading. On the other hand, constitutive activation of type I IFN and ISGs by chronic endogenous triggers could establish a state of exhaustion, as we observed in aged mice (22), and explain reduced responses to viral infections (46).

In conclusion, this study identifies WASp-mediated regulation of endosomal maturation and intracellular trafficking as a central mechanism for restraining activation of innate sensors, and brings forward the contribution of the STING pathway to excessive production of type I IFN inflammation in WAS.

## Methods

### Mice.

WASp^–/–^ mice on a C57BL/6 (CD45.2) genetic background were a gift from S. Snapper (Massachusetts General Hospital, Boston, Massachusetts, USA). Mice were bred and maintained in sterile isolators. Experiments were performed using homozygous WASp^–/–^ females or males as WKO mice and WT littermates as controls.

### Primary cell culture.

Mouse BM-derived DCs (BMDCs) were generated in vitro from BM of C57BL/6 WT or WKO mice using GM-CSF. DCs were cultured at a concentration of 1.5 × 10^6^ cells/mL in Nunc nontreated 6 well-plates (Thermo Fisher Scientific) for 7 days using IMDM supplemented with 10% FBS, 50 μM 2-mercaptoethanol (Gibco), 1% gentamicin, complemented with 30% supernatant of GM-CSF produced from J558 cell line. Cells were used for experiments between days 6 and 8. CD11c^+^ DCs were enriched from total spleen cells using the CD11c microbeads, mouse (Miltenyi Biotec).

Mouse BM-derived macrophages (BMDMs) were generated in vitro from BM of C57BL/6 WT or WKO mice using M-CSF. BM-derived cells were cultured at a concentration of 1 × 10^6^ cells/mL in non-treated 10 cm Petri dishes (Thermo Fisher Scientific) for 7 days using L929 conditioned medium: RPMI 1640 supplemented with M-CSF (30% L929 cell supernatant), 10% FBS, plus 1% penicillin/streptomycin, 1 mM sodium pyruvate. Cells were used for experiments between days 6 and 8.

L929 cells (M-CSF–producing cells) were grown for 1 week inRPMI 1640–supplemented 10% FBS plus 100 μg/mL penicillin, 100 μg/mL streptomycin, 2 mM l-glutamine, 10 mM HEPES, 1 mM sodium pyruvate, and 50 μM β-mercaptoethanol. Cell-free supernatant was then harvested from the confluent monolayer, 0.22 μm filtered, and kept at –20°C until use.

### Virus production and cell infection.

The expression plasmid and relative packaging with envelope were cotransfected into HEK293T cells using Lipofectamine 3000 (Invitrogen L3000-015). Twenty-four hours after transfection, the supernatant containing virus was collected, filtered, and added to cells for infection. A total of 3 × 10^5^ cells to be infected were dispensed in 1 mL per well in a 12-well plate and infected with virus by spinoculation at 1500 *g* for 60 minutes in the presence of Lipofectin (0.1%, Invitrogen). After spinoculation, cells were diluted by addition of 1.5 mL and incubated overnight. One day after infection 1.5 mL media was removed and replaced with 1.5 mL fresh cell culture medium supplemented with the relative selected antibiotic for resistance.

### Retroviral and lentiviral plasmids.

Plasmids were as follows: SEWW (EGFP-WASp) (47), Lenti CrisprV1 (Addgene 48535), Lenti CrisprV2 (Addgene 52961), MSCV herad3HAHOXB8 (29), and MSCV2.2 TLR9-HA (IRES-GFP) (48).

### sgRNA design.

sgRNAs for WASp and STING genome editing were designed using Genetic Perturbation Platform (GPP) sgRNA Designer (https://portals.broadinstitute.org/gpp/public/analysis-tools/sgrna-design) to minimize potential off-target effects. For WASp genome editing, 3 different WASP sgRNA sequences were cloned in a Lenti CrisprV1 plasmid previously digested with BsmBI restriction enzyme. For STING, only 1 previously published sgRNA (49) was cloned in Lenti CrisprV2 plasmid.

All sequences are reported as 5′→3′: WASp1 for CACCGGAATGTTCTGCTGAACGGC, rev AAACGCCGTTCAGCAGAACATTCc; WASp2 for CACCGGATGAAGTAGGACTTCTGA, rev AAACTCAGAAGTCCTACTTCATCC; WASp3 for CACCGAGCAAAAGTGTGGAAGAACG, rev AAACCGTTCTTCCACACTTTTGCTc; STING1 for, CACCGTCCAAGTTCGTGCGAGGCT, rev AAACAGCCTCGCACGAACTTGGAC.

### Generation of Hoxb8 progenitors cell lines and differentiation in DCs.

The method was previously described (29). Briefly, BM cells were collected from femurs of 4- to 8-week-old mice and separated on a Ficoll gradient. Cells were resuspended in RPMI containing 15% FBS and supplemented with 30% GM-CSF–containing supernatant plus 15% of Flt3L-containing supernatant. After 2 days of cell culture, cells were collected and resuspended in progenitor outgrowth medium (POM): RPMI supplemented with 10% FBS, 1% gentamicin, and 1 μM β-estradiol (MilliporeSigma, E-2758) and infected with MSCV herad-3HAHOXB8 retroviral particles. For 1 month cells were dispensed every 3–4 days in fresh medium and transferred into new wells. After this period, cells were expanded in Hoxb8 Outgrowth Medium (POM supplemented with 15% Flt3L supernatant). For subsequent differentiation experiments, cells were washed twice with warm PBS containing FBS and resuspended in a concentration of 0.5 × 10^5^ cells/mL in IMDM containing 30% GM-CSF.

### Cell stimulation.

DNA-ICs were formed in vitro by combining a monoclonal anti-DNA antibody (clone E11) with pLIT-CG50.1.plasmid (28), provided by MedImmune. Briefly, 100 μg of the E11 antibody was mixed to 10 μg of DNA plasmid and incubated at 37°C for 30 minutes. The IC preparation was diluted in medium to stimulate cells with the indicated concentrations. Cells were incubated for 3 hours for RNA extraction and RT-PCR or for 16 hours for protein analysis.

dsDNA sequences were purchased from Integrated DNA Technologies and were annealed at 200 μM strand concentration in NEB buffer 2 (B7002S), with incubation at 95°C for 100 seconds, followed by slow cooling at 25°C (50): 5′-TACAGATCTACTAGTGATCTATGACTGATCTGTACATGATCTACA; 5′-TGTAGATCATGTACAGATCAGTCATAGATCACTAGTAGATCTGTA.

CpGB (Invivogen, tlrl-1668), DMXAA (Invivogen, tlrl dmx), LPS (Invivogen, tlrl-pb5lps), cGAMP (Invivogen, tlrl-nacga23) were all used at the indicated concentrations (3 hours for RT-PCR, 16 hours for protein analysis). For dsDNA and cGAMP, used concentration were mixed with Lipofectamine 3000 (Invitrogen, L3000-015) following the suggested protocol.

### Flow cytometry.

For cell staining, FcR binding sites were blocked by using αCD16/CD32 (BioLegend, 93). Samples were then stained with specific antibodies in PBS plus 1% BSA and fixed with PBS plus 1% PFA. For intracellular staining, cells were fixed and permeabilized using Cytofix/Cytoperm solution (BD Biosciences) following manufacturer’s instructions, and then stained with specific antibodies. Flow data were acquired with a FACSCelesta or FACSAria II (BD Biosciences) and analyzed with Diva software (BD Biosciences) or FlowJo software (Tree Star Inc.). Antibodies used were WASp (Santa Cruz Biotechnology Inc., B-9) LIVE/DEAD Fixable Dead Cell Stain (Life Technologies, L34965).

### Recycling assay (Tf).

Cells were starved 15 minutes in media without FBS at 37°C; then cells were pulsed for 1 hour at 37°C with Tf 647 (50 μg/mL; Molecular Probes) to allow cells to reach equilibrium. Cells were moved to 4°C, washed twice in cold PBS, and treated for 2 minutes with cold acid buffer (glycine 50 mM, NaCl 150 mM pH 3), and the reaction was stopped by addition of 1 mL cold neutralization buffer (PBS, BSA 0.05%). Cells were then incubated at 37°C with nonlabeled Tf (50 μg/mL; Molecular Probes) for the indicated times and analyzed by FACS. Time 0 represents collection of cells after neutralization.

### Degradation assay (DQ-BSA).

Cells were pulsed for 10 minutes at 37°C with medium containing Alexa Flour 647–BSA (5 μg/mL; Molecular Probes). After Alexa Flour 647–BSA pulse, cells were washed twice with cold PBS in order to remove nonspecific binding BSA from the cell surface. Cells were then acquired on a FACS Aria II in real time at 37°C, and after the first minute cells were pulsed with DQ Green BSA (5 μg/mL; Thermo Fisher Scientific), then acquired for 45 minutes at 37°C. DQ-BSA lysosomal degradation was measured by flow cytometry as AUC, normalized on AF647-BSA^+^ cells.

### Western blot analysis.

Cells were washed and lysed with buffer containing 50 mM Tris-HCl (pH 7.6), 150 mM NaCl, 0.1% SDS, 1% NP-40, and protease/phosphatase inhibitors. Protein concentration was determined by Bradford Pierce BCA protein assay (Thermo Fisher Scientific) according to the manufacturer’s instructions. The supernatants were boiled for 10 minutes and separated by SDS-PAGE. Polyacrylamide gels were cast with GERBU acrylamide M-Bis 30% solution; depending on size, proteins were spread on running gel from 10% to 12%.

Primary antibodies included WASp (Santa Cruz Biotechnology Inc., B-9), phospho–NF-κB p65 (Cell Signaling Technology, 93H1), total NF-κB p65 (Cell Signaling Technology, C22B4), p-TBK1 (Cell Signaling Technology, 52C2), total TBK1 (Cell Signaling Technology, D52C2), STING (Cell Signaling Technology, D2P2F).

Secondary antibodies included anti-rabbit HRP and anti-mouse HRP (Jackson ImmunoResearch Laboratories Inc.).

### Real-time PCR.

Total RNA was extracted from DCs with TRIzol reagent (MilliporeSigma) according to the manufacturer’s instructions. cDNA was synthesized using SuperscriptII reverse transcriptase (Invitrogen) or SuperScript VILO (Invitrogen). Real-time PCR for gene expression was performed using SoFast EvaGreen Supermix (Bio-Rad) using specific primers, as follows: Ifnb for TCAGAATGAGTGGTGGTTG, rev GACCTTTCAAATGCAGTAGATTCA; Wash for AGGTGGGGACTTGATGTCAG, rev AGAGAAGGCTCCTCCAGGTC; Wasp-1 for GATATCGGAGCACCGAGTGG, rev GCAGATCCGGGTCTAGGTTG; Whamm for AGAGACATGCGAGAAGTTGC, rev CCTTCTAGGACCCAGCTCAT; N-wasp for AGGGTCACCAACGTGGGC, rev GGTGTGGGAGATGTTGTTG; Isg15 for AGCAATGGCCTGGGACCTAAA, rev CAGACCCAGACTGGAAAGGG; Mx1 for TCATCAGAGTGCAAGCGAGG, rev TCTGATACGGTTTCCTGTGCT; Oas1a for GGTCCAGAGTTCATGGTGGC, rev AACATGACCCAGGACATCAAAGG; Ifit2 for GGCCTTCTGCAGTTAATGCTT, rev TGTGCAGCACCTCTAAGTCTCAT; Gusb for ACTGACACCTCCATGTATCCCAAG, rev CAGTAGGTCACCAGCCCGATG.

### Quantification of type I IFN and inflammatory cytokines.

ELISA: 2 × 10^5^ cells were stimulated with CpG-B (1 μg/mL) for different times, and cytokine levels in cell culture supernatants were assessed by ELISA. IL-12p70 and TNF were detected by ELISA Max Standard sets (BioLegend), following the manufacturer’s instructions. The absorbance was read at 450 nm with an iMark Microplate Absorbance Reader (Bio-Rad). Soluble cytokines were measured in the supernatant of BMDMs using a bead-based immunoassay (Cytometric Bead Array [CBA], eBioscience). Fluorescence was read using an LSR II flow cytometer (BD Biosciences). Type I IFN was dosed using a biological assay based on the reporter B16-Blue IFN-α/β cells (InvivoGen). Cells were stimulated with the various treatments, and the cell culture supernatant was collected for the assay according to the manufacturer’s instructions.

### Cellular imaging.

To track intracellular ICs, 10^5^ DCs were seeded on fibronectin-coated slides (10 μg/mL; MilliporeSigma) and allowed to adhere for 30 minutes at 37°C. The indicated concentrations of ICs were added to the slides and incubated for 7, 15, or 40 minutes at 37°C. Cells were gently washed with warm PBS and fixed (4% paraformaldehyde). ICs were detected using an AF594–conjugated Fab to the Fc portion of ICs. For the other markers, cells were incubated with the primary antibodies listed in the next paragraph (in PBS: BSA 0.2%, saponin 0,05%) for 2 hours at room temperature (RT). After 2 washes with PBS, slides were incubated with secondary antibody (in PBS: BSA 0.2%, saponin 0.05%) for 1 hour at RT. Slides were mounted using Fluoro-Gel II with Dapi (Electron Microcopy Science).

### Antibodies used for cell labeling.

Primary antibodies included the following: EEA1 (Santa Cruz Biotechnology, sc-6415), Rab7 (Abcam, ab 137029), Lamp1 (BD Pharmingen, 1D4B), GFP (Life Technologies, A11122). Secondary antibodies included the following: donkey anti-goat–AF555 (Invitrogen, A21432), goat anti-rat–AF488 (Invitrogen A11006), chicken anti-rabbit–AF488 (Invitrogen, A21441).

Other reagents included the following: AF594/Fab (Jackson ImmunoResearch Laboratories Inc., 309-586-003), A488 phalloidin (Life Technologies, A12379), AF555 phalloidin (Life Technologies, A34055), DQ Green BSA (Life Technologies, D12050), Tf-AF647 (Life Technologies, T23366), dextran-AF488 (Life Technologies, ID7178), WGA-AF647 (Life Technologies, W32466).

### Image acquisition and analysis.

Confocal images were acquired with a LSM 880 META reverse microscope (Zeiss) with a 63×/1.4 NA plan oil objective. The super-resolution images in Figure 2E and Figure 3, A and C, were acquired using the Airyscan detector and processed by online reassignment of the pixel information, followed by linear deconvolution. Image analyses were performed using Volocity 3D Image Analysis Software 5.5.1 (PerkinElmer) and Fiji, ImageJ plugin (NIH). For quantification of organelles, objects were identified using a cutoff of 0.1μm^2^, and vesicle areas were measured automatically by the Volocity software. To measure the distance of each endosome from the nucleus, the length of the segment between the centroid of the endosome and the centroid of the nucleus was calculated using ImageJ. Peri-endosomal WASp and F-actin distribution and density were quantified as depicted in the scheme in Figure 3B. To identify the peri-endosomal area, we computed a region spanning 0.8 μm from endosome boundaries. Endosomes were defined as EEA1-positive structures greater than 0.1 μm^2^. Then we applied a threshold to isolate F-actin and WASp signals from the background. Finally, we calculated WASp distribution as the percentage of its signal present in cytosol and in peri-endosomal area, and WASp and F-actin density as the ratio of signal above the threshold on the total area analyzed.

### Dextran-FITC assay to assess endosomal membrane permeability.

To analyze dextran leakage, cells were starved for 20 minutes in medium without FBS and pulsed with 200 μg/mL dextran-AF488 for 20 minutes. Cells were washed fixed and analyzed by confocal microscopy. To analyze the fraction of diffused cytosolic dextran, we optimized a preexisting macro to analyze images of dextran-FITC–labeled cells in a semiautomated fashion. The macro’s output was coupled with a function written in R language (R software version 3.4.2) for data mining and rearrangement. In brief, dextran signal was categorized as “compartmentalized” or “diffuse,” according to a stringent, manually chosen threshold of pixel intensity (of 0–255). Subsequently, green areas that were at first recognized as diffuse underwent a second round of filtering according to their surface and shape. Regardless of pixel intensity, green spots of 0.05–0.80 μm bearing a round coefficient greater than 0.40 were considered as “compartmentalized” signal in the final output.

### Electron microscopy.

DCs were incubated with BSA-Au^5^ conjugates (Cell Microscopy Core, UMC Utrecht) added to the medium for 2 hours at 37C° to mark endocytic trafficking route. Afterward, DCs were fixed at RT by addition of Karnovsky fixative (2.5% glutaraldehyde and 2% formaldehyde [Electron Microscopy Sciences] in 0.2 M phosphate buffer, pH 7.4) in equal amount to culture medium for 15 minutes. Then, the medium was replaced by fresh fixative for 2 hours at RT. DCs were then post-fixed with 1% OsO_4_/1.5% K_3_Fe(III) (CN)_6_ in 0.065 M phosphate buffer for 2 hours at 4°C and finally 1 hour with 0.5% uranyl acetate. After fixation, cells were dehydrated and embedded in epoxy Epon resin (Polysciences). Ultrathin sections of 60 nm were contrasted with uranyl acetate and lead citrate using AC20 (Leica) and examined with a Tecnai T12 transmission electron microscope (FEI Thermo Fisher Scientific). Images were taken from 3 different blocks of cells per condition. Quantification of electron microscopic images was done using Fiji. For quantification of the area and perimeter of endolysosomes, the smallest possible ellipse to surround the organelle was drawn in Fiji with the freehand selection tool, and then the ellipse was measured. Fifty random images per block were taken and analyzed (in total 3 × 50 = 150 cell profiles were measured per experimental condition).

### Live microscopy.

To mark endocytosis, 2 × 10^5^ DCs were seeded for 30 minutes in a Nikon chamber at 37°C with 5% CO_2_ on MatTek microwell dish. After adhesion, cells were pulsed for 5 seconds with 5 μg/mL WGA-AF647. After 2 washes in warm PBS, warm complete media was added. Cells were recorded in epifluorescence for 45 minutes with 1 frame every 15 seconds on a C1 Nikon reverse microscope with a 60× objective.

### Treatment with inhibitors.

Cells were treated with 25 μM of Arp2/3 inhibitor CK666 (Tocris Bioscience, 3950), Lat-A (0.5 μM; MilliporeSigma, L5163). Control cells were treated with an equivalent concentration of DMSO. After 30 minutes of incubation, cells were washed and used for the different experiments. The STING inhibitors C-176 (PC-35311) and C-178 (PC-35310) (34) were purchased from ProbeChem. The cGAS inhibitor RU.521 was purchased from Invivogen (inh-ru521). For in vitro treatments, cells were preincubated with 0.5 μM C-176, 50 μM RU.521, or the equivalent concentration of DMSO. After 30 minutes, stimuli were added to the wells without washing the inhibitor. RNA or cell culture supernatant was collected after 3 and 16 hours, respectively. For in vivo treatment, mice were injected with 7.5 μL C-176 or DMSO dissolved in 85 μL corn oil twice per day for 11 consecutive days. Mice were euthanized, and total spleen was harvested. RNA was extracted from single-cell suspension to perform RT-PCR.

### Statistics.

All statistical analyses were performed using GraphPad Prism 6 software. All data are reported as mean ± SEM. Unpaired 1-tailed Student’s *t* test or 2-way ANOVA was used to assess significance. *P* values less than 0.05 were considered significant.

### Study approval.

The study was approved by International Centre for Genetic Engineering and Biotechnology (ICGEB) board for animal welfare and authorized by the Italian Ministry of Health (Aut. N.1155/2016-PR). Animal care and treatment were conducted with national and international laws and policies (European Economic Community Council Directive 86/609; OJL 358; December 12, 1987). Mice were housed and handled according to institutional guidelines and experimental procedures approved by the International Centre for Genetic Engineering and Biotechnology (ICGEB) board. All experiments were performed in accordance with Federation of European Laboratory Animal Science Association (FELASA) guidelines.

## Author contributions

GMP and AN performed key experiments, analyzed data, prepared figures, and contributed to manuscript writing. GS, RA, NC, KECL, AC, HA, and FG performed extra experiments. NL and JK performed and analyzed electron microscopy experiments. PB provided conceptual support and data interpretation. HH and RNA provided reagents. FB ideated and supervised the study and wrote the manuscript.

## Supplementary Material

Supplemental data

Supplemental Video 1

Supplemental Video 2

## Version 1 07/28/2020

In-Press Preview

## Version 2 09/03/2020

Electronic publication
