# Supplementary material for: Wiskott-Aldrich syndrome protein restricts cGAS/STING activation by dsDNA immune complexes
Source: JCI Insight. 2020 Sep 3;5(17):e132857. doi: 10.1172/jci.insight.132857 (PMC7526445; doi:10.1172/jci.insight.132857)
Supplement: Supplemental data [file jciinsight-5-132857-s195.pdf]

Figure S1. Cytokine responses in DCs and macrophages

A-B) DC were stimulated with graded doses of CpG-B for different time points. *Ifnb* transcription was evaluated by RT-PCR (A). IFN-β, TNF and IL-12p40 protein secretion was evaluated by ELISA after 16hrs (B). **C)** Bone marrow derived macrophages were stimulated with 1µg/ml of CpG-B for 16hrs. IL-6 IL-12p40 and TNF protein secretion was evaluated by cytometric bead array (CBA).

Figure S2. Generation of Hoxb8 immortalized DCs progenitors.

Schematic representation of the two steps procedures to generate immortalized DCs precursors according to the protocol described in (28). First fresh bone marrow cells are infected with retroviruses expressing an estrogen-regulated form of Hoxb8 and exposed to Flt3L to promote commitment to early myeloid progenitors. Upon removal of estrogen from the medium and exposure to GM-CSF cells can be differentiated into DCs. Commited progenitors have been infected with a lentivirus encoding CAS9 and sgRNA guides to WAS. Cells were selected by puromycin and single cell sublclones were screened for expression of WASp by Flow cytometry. Clones expressing WASp (WT^{CAS}) and clones deleted for WASp expression WKO^{CAS} were selected from the same plate of subcloning and used in subsequent experiment.

Figure S3. Aberrant DNA-IC degradation in WKO cells

A) Cells were pulsed with 90 µg/ml of ICs and lysed after 20, 45 or 90 min. Whole cell lysates were developed with antibody to IC and anti-Gapdh antibody as loading control. The blots show one representative of 3 experiments.

Figure S4. Altered endocytosis and endo-lysosomes distribution in WKO cells
A) WT and WKO DCs were labelled with antibodies to EEA-1. The distance between each individual endosome and the nucleus center of mass was calculated on 25 cells/genotype. **B)** Electron microscopy of DCs loaded with BSA-gold (Epon embebbed sample). Images are representative examples of endosomes in WT and WKO DCs. Black arrows point to aberrant endosomes. Bars show quantification of area, diameter and perimeter of endosomes. Data were acquired on 150 cell/genotype from two independent experiments. Significance was determined by unpaired t test with $p<0.05$** $p<0.001$***, $p\leq0.0001$****. **C)** Single frames extracted from supplementary Movie 1 and 2. Cells were loaded with A647-WGA, washed and immediately recorded for 40 min to visualize early events of endocytosis. Frames were acquired every 15s.

Figure S5. Expression of nucleation promoting factors (NPFs) in DCs and effect of the Arp2/3 and actin inhibitor on endosomes size and DNA-IC intracellular trafficking.
A) The expression levels of 4 different NPFs of the WAVE/WASp family were evaluated by RT-PCR in DCs and mouse embryonic fibroblasts (MEF), as control. Values are means ± SEM of two independent experiments. **B)** WT DCs were treated for 30 min with the Arp2/3 inhibitor CK666 (25µM) Lat-A (0,5 µM) or DMSO as control. Cells were stained with antibody against EEA-1 and phalloidin to visualize F-actin. Images show representative single confocal planes (scale bar 10 µm). Endosomes area was quantified on individual cells, data were collected from 25 cells/ condition from 2 independent experiments, bars show means ± SEM of endosomes area. Significance was determined by one way ANOVA, $p<0.01$**, $p\leq0.0001$****. **C)** WT DCs were pre-treated with CK666 or control vehicle (DMSO) for 30 min, washed and pulsed with 90 µg/ml IC for 7 min, followed by 40 min of chase. Representative examples show the relative distribution of IC, EE and lysosomes (LAMP-1) and the corresponding co-localization mask. Insets are magnification of the region depicted by dotted line. Scale bars is 5 µm and 1.5 µm for insets. **D)** The graphs show quantification of the area of intracellular DNA-IC structures, MOC IC/EEA1 and MOC IC/Lamp1 after 40 min of chase. All data are mean ± SEM of at least 26 cells/condition from 2 independent experiments, significance was determined by unpaired t test ***$p\leq0.001$. **E)** Transcriptional level of *Ifnb* in stimulated (DNA-IC 90 µg/ml) WT DCs pre-treated with CK666 or DMSO for 30 min. Graph show the mean ± SEM of 4 biological replicate. Significance was determined unpaired t test *$p\leq0.05$.

Figure S6.
A) WT and WKO DC were stimulated with various concentrations of DMXAA. Type-I IFN protein was evaluated by B16 BLUE IFN reporter assay. Graph show means ± SEM of 3 independent experiments **B)** Titration of the STING inhibitor C178 on WT and WKO cells. The levels of IFN protein induced by DMXAA (3µg/ml) in cells pre-treated with graded dose of C-178 or vehicle (DMSO) were evaluated by B16-BLUE reporter assay. One representative of two experiments. **C)** Western Blot of phosphorylated TBK1 (p-TBK1) and total TBK1 in DCs treated with DMSO or C- 178 and stimulated with 5µg/ml of DMXAA (one representative of 2 biological replicate).

D) Schematic representation of the procedure to generate WKO-STING KO DCs precursors. WKO Hoxb8 cells have been infected with a lentivirus encoding CAS9 and sgRNA guides to STING or control. Cells were selected by puromycin and used in subsequent experiments. **E)** Western Blot showing full deletion of STING in WKO-STING KO. One representative of two experiments. **F)** Transcriptional level of *Isg15* in WKO treated with RU.521 (50 μM) or vehicle (DMSO) and stimulated with DNA-IC (90 μg/ml). Data are expressed as ratio between inhibitor treated and DMSO treated cells. Significance was determined by unpaired t- test. Graphs show the mean ± SEM of 6 biological replicate.

Supplementary Movie 1 and 2.
Time-lapse epifluorescence microscopy of WGA internalization to visualize endocytosis in WT (1) and WKO (2) BMDCs. Movie started 1 min after the WGA pulse. Frames were acquired every 15s for 40 min.

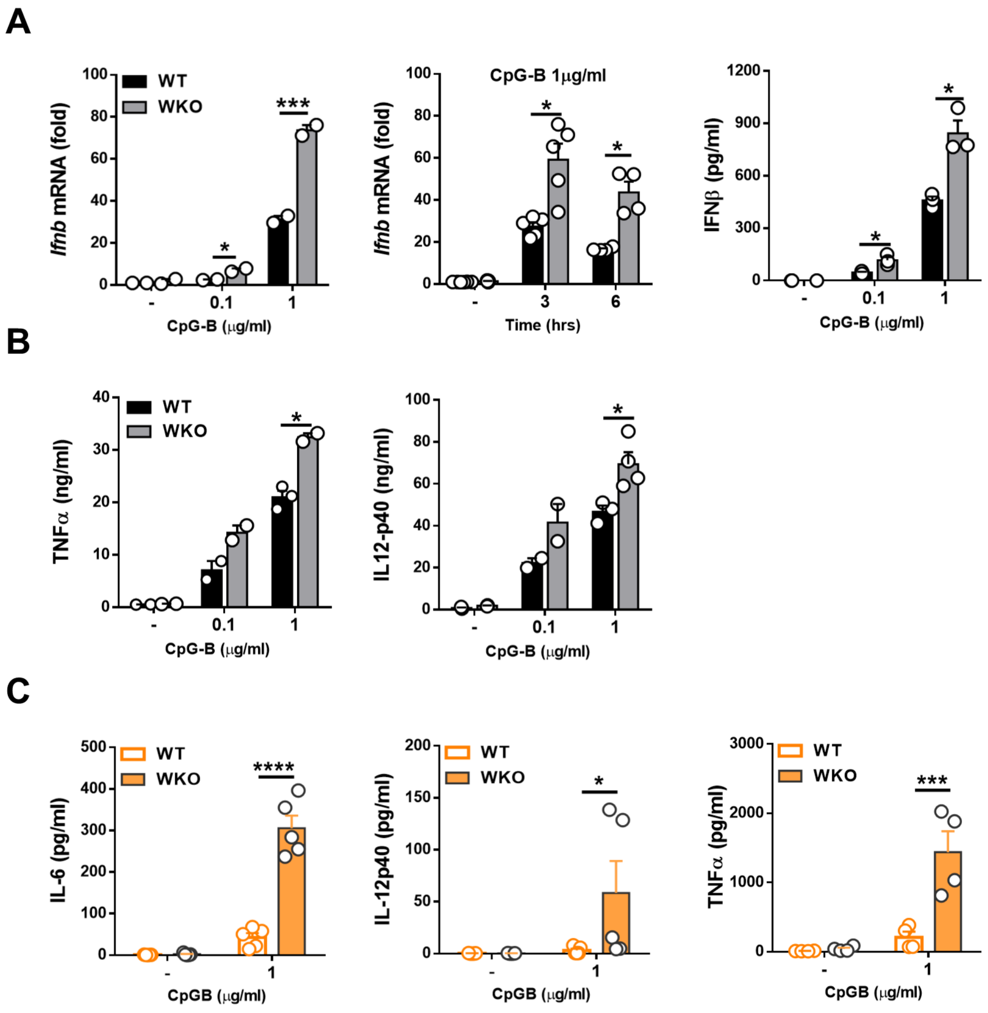
A
B
C
Ifnb mRNA (fold)
WT
WKO
CpG-B (µg/ml)
CpG-B 1µg/ml
Time (hrs)
IFNβ (pg/ml)
TNFα (ng/ml)
IL12-p40 (ng/ml)
IL-6 (pg/ml)
IL-12p40 (pg/ml)
CpGB (µg/ml)
*
***
****

Figure S1

A

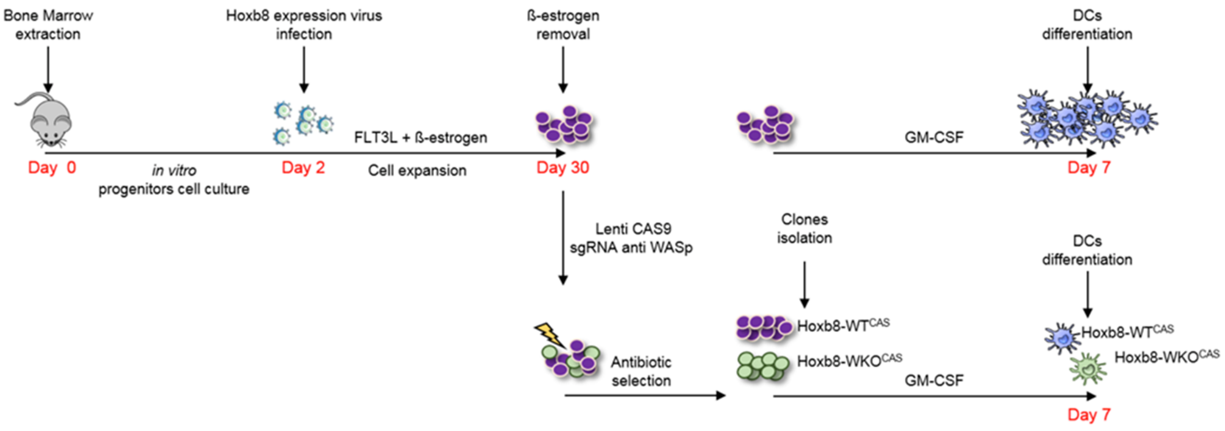

Figure S2

A

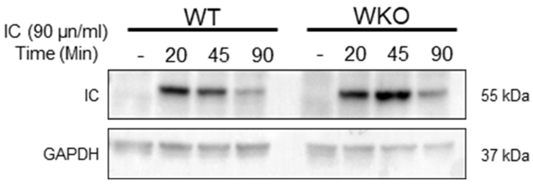

Figure S3

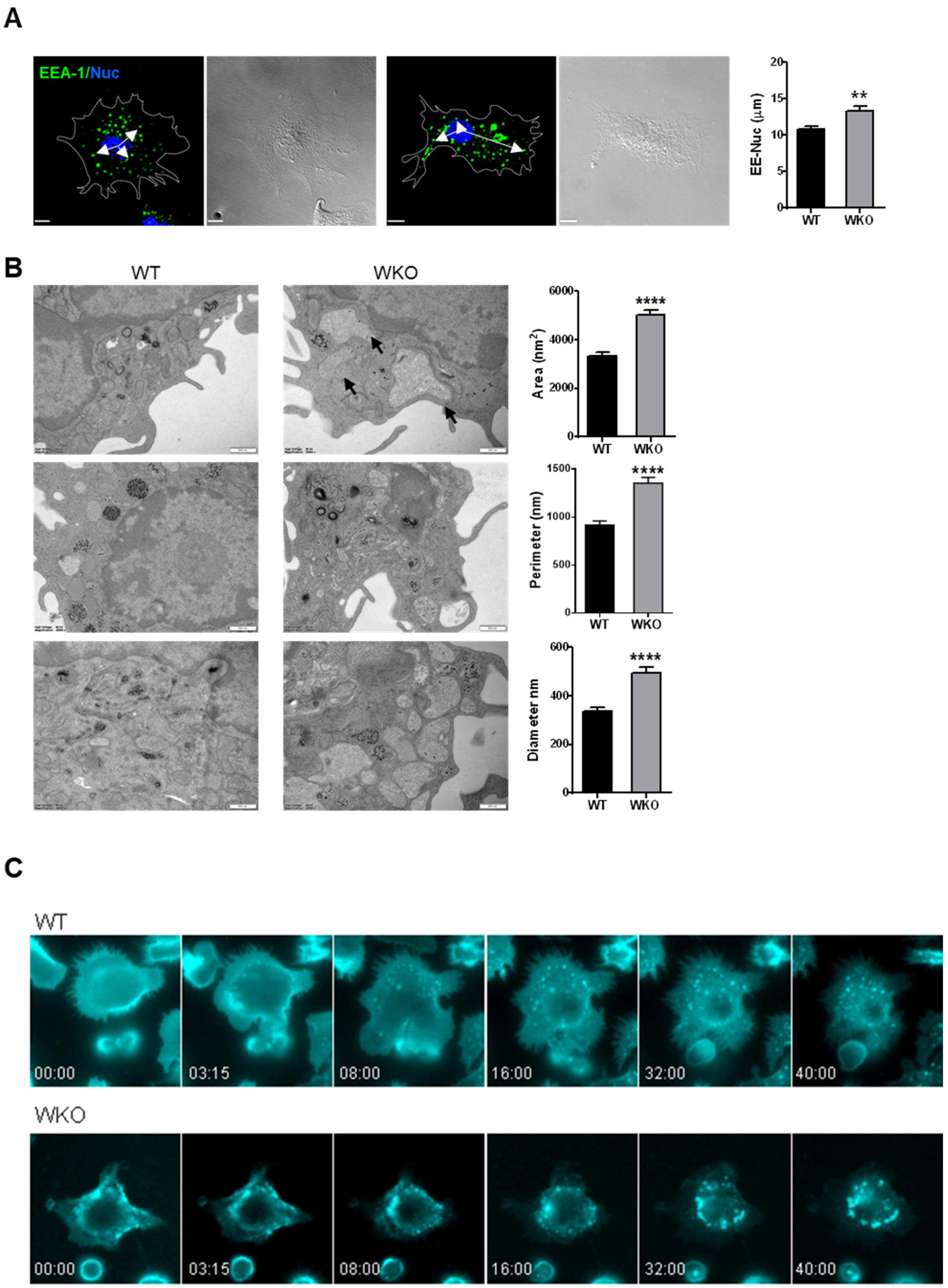
A
EEA-1/Nuc
EE-Nuc (μm)
20
15
10
5
0
WT
WKO
**
B
WT
WKO
Area (nm2)
6000
4000
2000
0
****
Perimeter (nm)
1500
1000
500
0
****
Diameter nm
600
400
200
0
****
C
WT
00:00
03:15
08:00
16:00
32:00
40:00
WKO
00:00
03:15
08:00
16:00
32:00
40:00

Figure S4

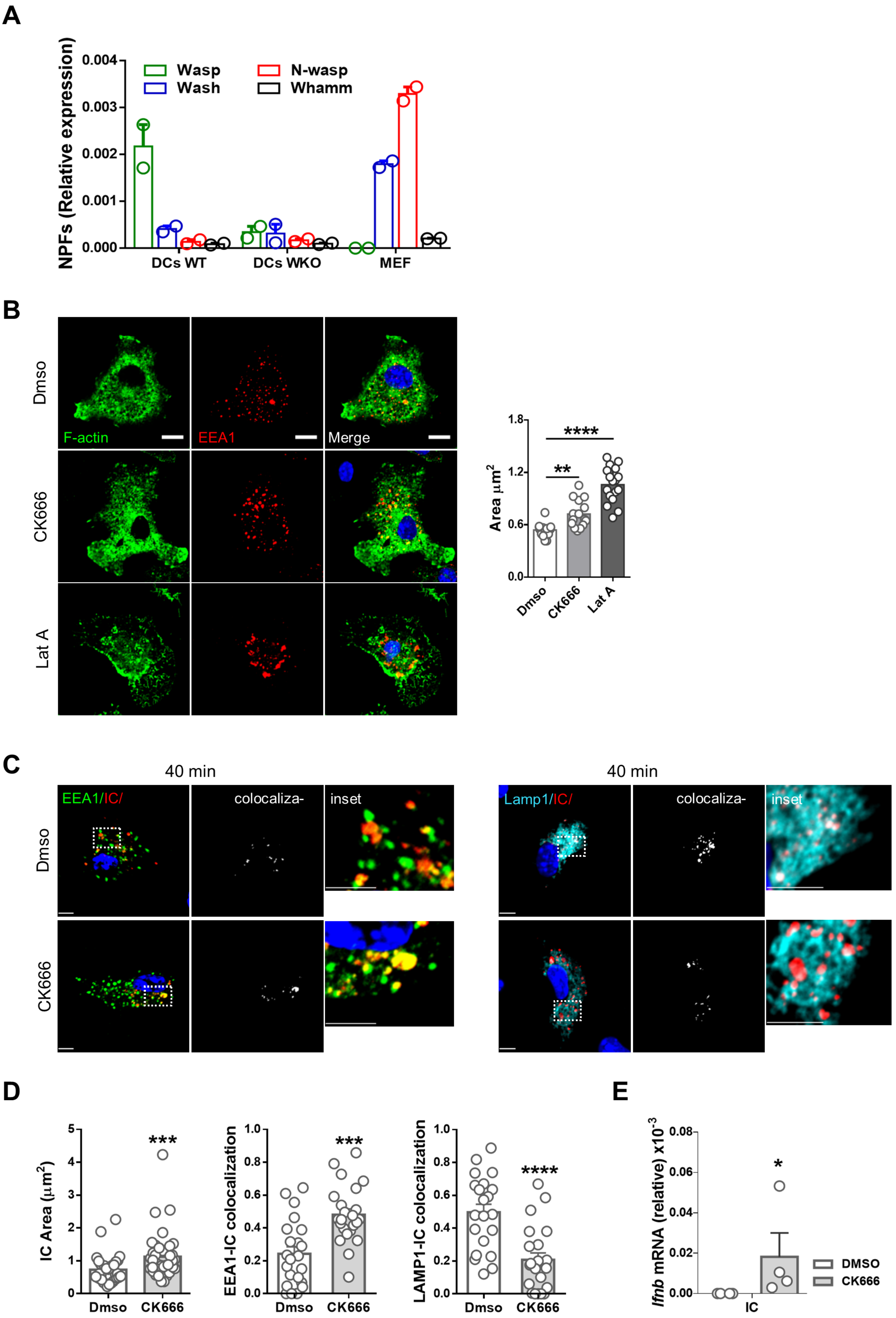
A
NPFs (Relative expression)
0.004
0.003
0.002
0.001
0.000
Wasp
Wash
N-wasp
Whamm
DCs WT
DCs WKO
MEF
B
Dmso
CK666
Lat A
F-actin
EEA1
Merge
Area µm2
1.8
1.2
0.6
0.0
****
**
Dmso
CK666
Lat A
C
40 min
EEA1/IC/
colocaliza-
inset
Lamp1/IC/
Dmso
CK666
D
IC Area (µm2)
5
4
3
2
1
0
***
Dmso
CK666
EEA1-IC colocalization
1.0
0.8
0.6
0.4
0.2
0.0
***
LAMP1-IC colocalization
****
E
Ifnb mRNA (relative) x10-3
0.08
0.06
0.04
0.02
0.00
*
IC
DMSO
CK666

Figure S5

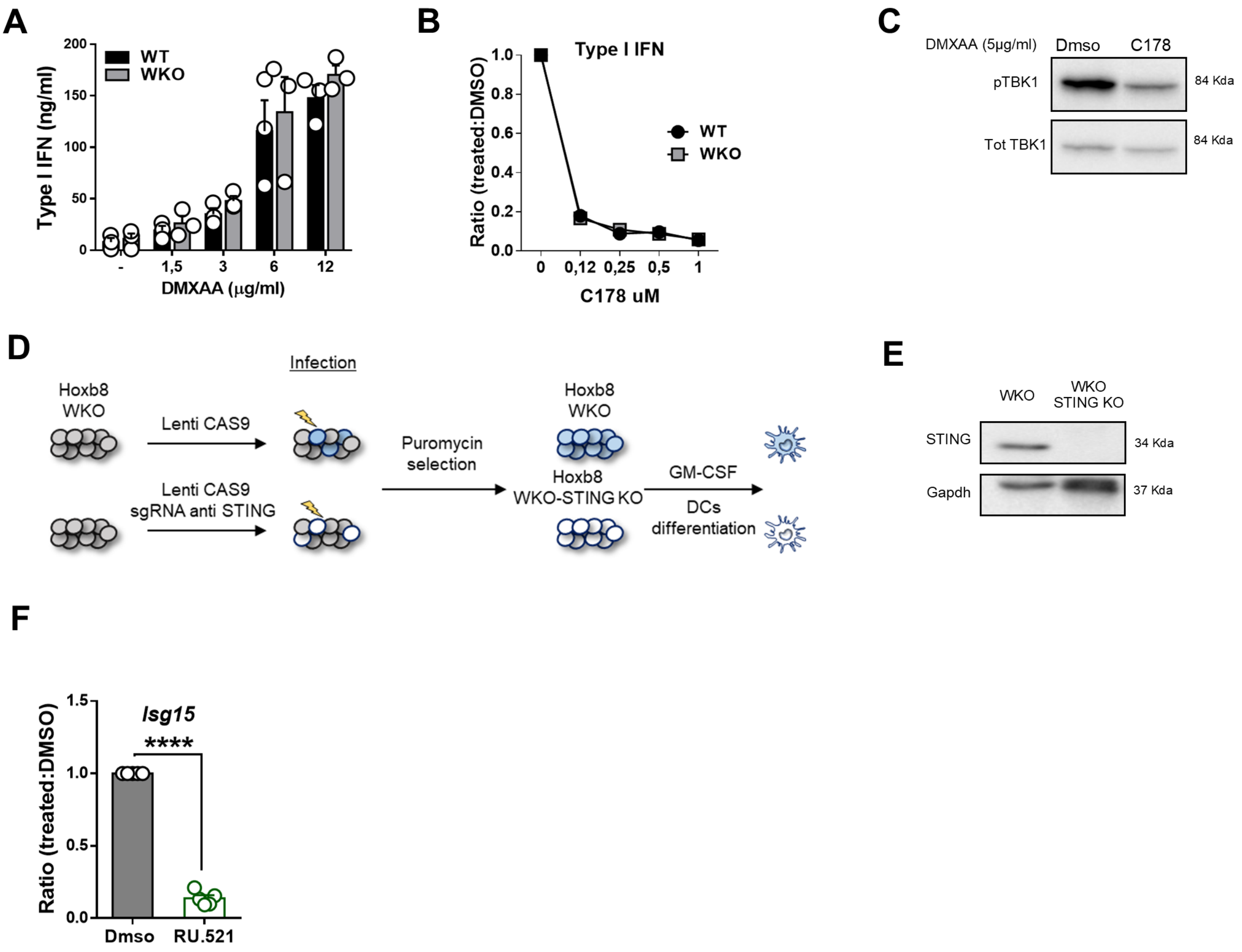
A
Type I IFN (ng/ml)
WT
WKO
DMXAA (μg/ml)
B
Type I IFN
Ratio (treated:DMSO)
C178 uM
C
DMXAA (5μg/ml)
Dmso
C178
pTBK1
Tot TBK1
84 Kda
D
Hoxb8 WKO
Lenti CAS9
Lenti CAS9 sgRNA anti STING
Infection
Puromycin selection
Hoxb8 WKO-STING KO
GM-CSF
DCs differentiation
E
WKO STING KO
STING
34 Kda
Gapdh
37 Kda
F
Isg15
Ratio (treated:DMSO)
Dmso
RU.521

Figure S6
